# Water Quality of Inflows to the Everglades National Park over Three Decades (1985–2014) Analyzed by Multivariate Statistical Methods

**DOI:** 10.3390/ijerph15091882

**Published:** 2018-08-30

**Authors:** Lei Wan, Xiaohui Fan

**Affiliations:** 1 Xuzhou University of Technology, No. 2 Lishui Road, New City District, Xuzhou 22111, China; hjwanl@163.com; 2 Soil and Water Science Department at Tropical Research and Education Center, IFAS, University of Florida, 18905 SW 280th Street, Homestead, FL 33031, USA

**Keywords:** water quality, inflows to everglades, trend analysis, CA, PCA

## Abstract

The Everglades, a vast subtropical wetland, dominates the landscape of south Florida and is widely recognized as an ecosystem of great ecological importance. Data from seven inflow sites to the Everglades National Park (ENP) were analyzed over three decades (1985–2014) for temporal trends by the STL (integrated seasonal-trend decomposition using LOESS) method. A cluster analysis (CA) and principal component analysis (PCA) were applied for the evaluation of spatial variation. The results indicate that the water quality change trend is closely associated with rainfall. Increasing rainfall results in increasing flow and thus, decreasing concentrations of nitrogen and phosphorus. Based on 10 variables, the seven sampling stations were classified by CA into four distinct clusters: A, B, C, and D. The PCA analysis indicated that total nitrogen (TN) and total phosphorus (TP) are the main pollution factors, especially TN. The results suggest that non-point sources are the main pollution sources and best management practices (BMPs) effectively reduce organic nitrogen. However, TN and TP control is still the focus of future work in this area. Increasing the transfer water quantity can improve the water quality temporarily and planting submersed macrophytes can absorb nitrogen and phosphorus and increase the dissolved oxygen (DO) concentration in water, continuously improving the water quality.

## 1. Introduction

Surface water, especially from rivers and lakes, is closely related to human activities, so the water quality cannot be ignored. Congress created the Everglades National Park (ENP) in 1947 to protect and preserve portions of the south Florida ecosystem. Because the Everglades is located in the upstream region of the ENP, the area is subject to upstream water management practices. In order to protect and monitor the ENP waters, Water Conservation Areas (WCAs) and Stormwater Treatment Areas (STAs) were constructed to improve the water quality in the north of the ENP.

Some researchers have focused on water quality improvement in WCAs and STAs. For example, Entry [1] studied the impact of STAs and best management practices (BMPs) on nutrient concentration in the Florida Everglades using Kendall tau or Tobit trend analyses for the entire year. The trend analyses suggested that agricultural BMPs have led to a large initial reduction in nutrient concentrations in the ENP. Chen et al. [2] examined annual phosphorus (P) removal in the six Everglades STAs during 1995–2011. STA performance, in terms of outflow total phosphorus (TP) concentration, was shown to depend on three primary variables: the hydraulic loading rate (HLR), the inflow TP concentration, and the loading rate (PLR). However, the mechanism through which these modifications have affected water quality in the ENP remains unknown. Long-term studies have investigated how to evaluate water quality trends, and identify indicators of water quality characteristics and laws of succession. Traditionally, the water quality change trend has been analyzed with linear regression analysis or nonparametric methods [3,4,5,6]. However, linear or monotonic trends cannot show the direction of intermediate reversals [7]. Due to human disturbance and other natural factors, water quality is not assured with linear and monotonic assumptions. To solve this problem, seasonal trend decomposition using the LOESS (STL) method is applied to water quality evaluation, which is one way to deal with the nonlinear and nonparametric statistical methods of local trends [8]. The STL method was first proposed and applied to the trend analysis of atmospheric CO_2_ concentration and the unemployed population in the United States by Cleveland [9]. In the water quality trend analysis, Qian et al. [7] presented the spatial and temporal trends of nutrient concentrations in the Neuse River and Estuary, North Carolina, USA with the STL methodology. The results showed that the shift from nitrogen to phosphorus limitation induced a change in the biotic community that explained the perception of worsening eutrophication [7]. Liang et al. [8] employed STL to identify the water quality trends in Lake Dianchi, China. Stow et al. [10] determined long-term trends and seasonal patterns in Maumee River rainfall, discharge, nutrient concentrations, and loads. The STL approach identified nonlinear trends and seasonal interactions that would be missed by traditional trend detection methods [7].

Recently, multivariate statistical tools have been applied to analyze the variations, relations, and distributions of the water quality data. A multivariate statistical analysis was also conducted to evaluate water quality in the inflow and outflow to the Everglades National Park in this paper. Cluster analysis (CA) and principal component analysis (PCA) are the most commonly used multivariate statistical methods in environmental studies [11,12,13,14]. The combined use of CA and PCA classified water samples into different groups based on their hydrochemical characteristics [13].

Hanlon et al. [15] and Fan et al. [16] studied the water quality of inflows to the Everglades National Park from 1977 to 2005 with statistical methods. Fan et al. [16] mainly analyzed water temperature, turbidity, pH, specific conductivity, dissolved oxygen, color, and alkalinity without nitrogen and phosphorus parameters with the Kolmogorov–Smirnov test and simple regression. The research results of Hanlon et al. [15] indicated that the overall ENP inflow water quality improved from 1977 to 2005, and total nitrogen (TN), total Kjeldahl nitrogen (TKN), total organic N, and total phosphorus (TP) concentrations generally decreased at inflow sites. This paper examined the time series of monthly values of selected physical and chemical water quality parameters and the discharges at the seven stations in the Everglades National Park (ENP) over the last three decades (1985–2014) with the STL method and PCA and CA. Compared with the research of [15,16], this research updated the time scale and analyzed the trend in water quality change with the STL method. The STL method includes a much wider range of component patterns than any single parametric method [17]. CA and PCA are helpful for streamlining stations and identifying the main pollution factors. The objectives of this study were (i) to evaluate the trends of physical and chemical parameters at seven inflow stations to the ENP over the last three decades and (ii) to search for impact factors on water quality change and provide suggestions for water quality managers.

## 2. Materials and Methods

### 2.1. Study Area and Data Source

**Figure 1:**
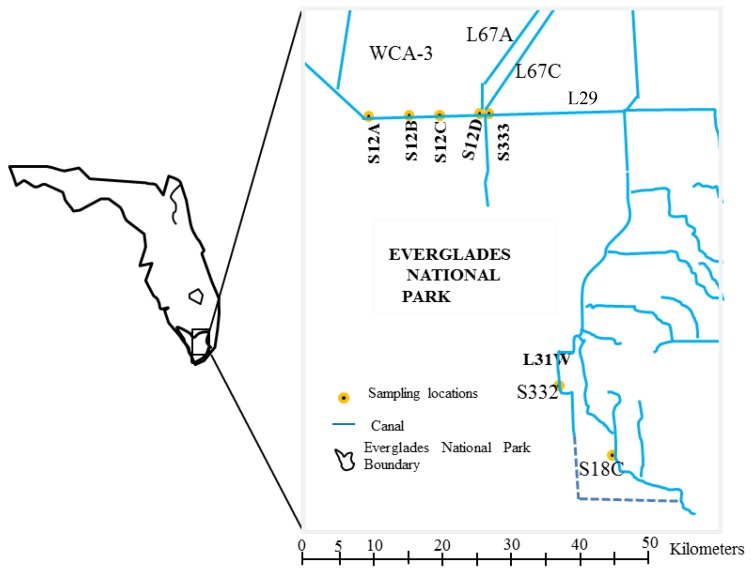
Location of inflow water quality monitoring stations to the Everglades National Park.

In this paper, selected chemical and physical water quality parameters were examined according to a monthly time series at seven stations at L29 and L31W canals in the ENP in the last three decades (1985–2014) (Figure 1; The relative position and functions of L29, L67C and L67A canals refer to http://www.evergladeshub.com/). Among the seven stations, S12D and S332 sites were cancelled after 2008 and 2006, respectively. Water quality parameters and hydrogeologic data were downloaded from the South Florida Water Management District’s database (SFWMD: http://www.sfwmd.gov/portal/page/portal/pg_grp_Sfwmd_era/pg_sfwmd_era_dbhydrobrowser). The monitoring sites are named S333, S332, S18C, S12A, S12B, S12C, and S12D based on their structures. Sample handling and chemical analyses were done in accordance with published protocols [18]. The detection limits and analytical methods for selected parameters are listed in Table 1. The statistical evaluations included dissolved oxygen (DO), pH, turbidity (TURB), total nitrogen (TN = TKN + NO_X_–N), ammonia nitrogen (NH_4_–N), total Kjeldahl nitrogen (TKN), total phosphorus (TP), ortho-phosphorus (PO_4_–P), and rainfall.

### 2.2. Seasonal Trend Decomposition Using the Loess (STL) Method

The time series of indicators were analyzed by seasonal trend decomposition using the Loess (STL) method. LOESS is considered to be a smoother and a nonparametric statistical method. In STL, observed time series are decomposed into the trend component, seasonal component, and residual component [7]. The trend component with low frequency can be considered to be a change tendency. The seasonal component with high frequency can be viewed as variation with stable seasonal disturbance. The residual component with random disturbance can be regarded as irregular variation. Therefore, removing the seasonal and residual components to obtain the trend component is very useful for understanding the change tendency of variables [19].

### 2.3. Cluster Analysis (CA) and Principal Component Analysis (PCA)

In this study, hierarchical cluster analysis (CA) and principal component analysis (PCA) were performed with the commercial statistics software package SPSS version 19.0 for Windows. CA is an extremely useful tool which can classify a set of observations into several mutually exclusive unknown groups based on a combination of internal variables. CA allowed the grouping of river water samples based on their similarities in chemical composition [20]. PCA is a very powerful technique for reducing the dimensionality of a data set with many inter-related variables [12]. An eigenvector is weighted linear combination of the original variables, which is equal to coefficients multiplied by the original correlated variables to obtain new uncorrelated (orthogonal) principal components. PCA allows easier interpretation of a given multidimensional system through reducing the number of correlated variables to a smaller set of orthogonal factors [21].

## 3. Results

### 3.1. Basic Statistics of the Monthly Measured Data at Each Station

The basic statistics of the monthly measured data on water quality are summarized in Table 2. DO was measured from 8:00 a.m. to 3:30 p.m. The maximum value of DO was 15.4 mg L^−1^ at S12A, and the minimum value was 0.1 mg L^−1^ at S332. The maximum values of DO at all seven stations exceeded 8 mg L^−1^ which shows very high primary productivity in the water. However, the minimum values of DO were only 0.1–0.6 mg L^−1^ which shows that the water was sometimes under the anaerobic condition. The magnitudes of the mean values at each station were in the following order: S12B > S18C > S12A > S12C >S12D > S333 > S332. The pH value was relatively stable across all stations in the range of 7.19–7.45. Turbidity values were relatively low, and the mean value was below 2 NTU, except at S332. The maximum concentration of NH_4_–N was 1.36 mg L^−1^ at S12B; the minimum value was below 0.005 mg L^−1^, and the mean value at all stations was below 0.1 mg L^−1^, except at S332. The mean TKN and TN values were followed the same order of magnitude: S333 > S12D >S12C > S12B > S12A > S332 > S18C. The PO_4_–P and TP concentrations were very low at all stations and higher at the stations on L29 than in L31W and C111. The water quality standard in the Everglades is 0.01 mg L^−1^ for TP (Florida Administrative Code, FAC 62-302.540) and 1.27 mg L^−1^ for TN [22]. The mean values of TN at S12C, S12D and S333 were 1.29, 1.37, and 1.44 mg L^−1^, respectively, which are higher than the standard value, and TP exceeded this criterion at all stations except S332 and S18C. The mean values of TP and PO_4_–P at both S332 and S18C in C111 were much lower than those at S333 in L31W.

### 3.2. Trend Analysis

#### 3.2.1. DO, pH, and Turbidity

**Figure 2:**
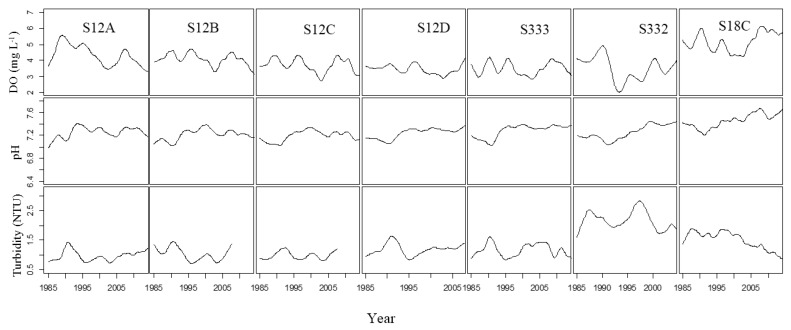
Trend changes of DO, pH, and turbidity at seven stations.

The trend changes in DO, pH, and turbidity at S12A, S12B, S12C, and S12D were similar (Figure 2), as were the DO trend changes at S332, S333, and S18C. There were upwards trends in DO from 1985 to 1990 and 2005 to 2010 and downwards trends in DO from 1995 to 2003 and after 2007 for S12A, S12B and S12C. The DO values were higher in 1990 and 1996 and lower in 1993 and 2003 at S12D. There was a very clear downward trend in DO values from 1990 to 1995 at all stations, especially at S332. On the whole, the pH values showed similar upwards trends at all stations with the lowest point reached in 1992, except for at S12A. A downwards trend in turbidity was found at S18C, although there were some fluctuations. All stations showed an obvious downwards trend in turbidity from 1990 to 1998 and an upwards trend at S12A, S12B, S12C, and S12D after 2005. Almost all stations had their minimum values at around 1995 and 2005.

#### 3.2.2. NH_4_–N, TKN, and TN

**Figure 3:**
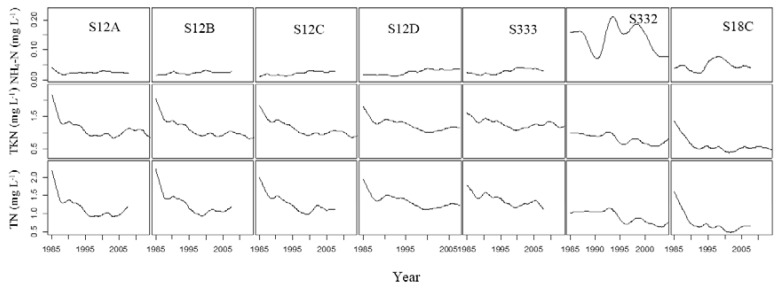
Trend changes in NH_4_–N, TKN, and TN at seven stations.

Figure 3 shows the change trends in NH_4_–N, TKN, and TN at the seven stations. There were similar change trends in NH_4_–N, TKN, and TN at S12A, S12B, S12C, S12D, and S333 in L29. There was no NH_4_–N change trend at S12A, S12B, S12C, or S12D, while there was an obvious downward change at S332 and S18C after 1999. The NH_4_–N concentration was obviously higher at S332 than at other stations. TKN and TN change trends were similar at S332 and S18C. There were downwards trends in TKN and TN at all stations over the three decades, but changes were slow after 1995. TN was lower from 1995 to 2005. There was almost no change in NH_4_–N at S12A, S12B, S12C, S12D, and S333, but TKN and TN decreased, indicating that organic nitrogen decreased at all stations

#### 3.2.3. PO_4_–P and TP

**Figure 4:**
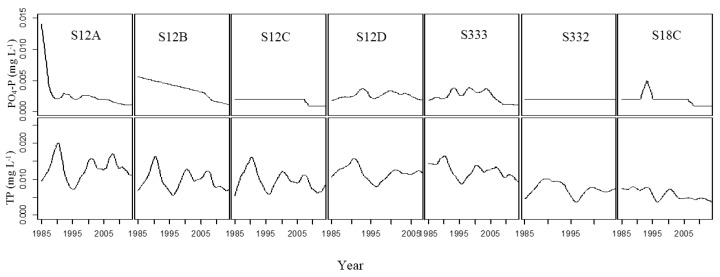
Trend changes in PO_4_–P and TP at seven stations.

There was a downwards Loess trend in PO_4_–P for all stations (Figure 4). The PO_4_–P concentration decreased drastically before 1990, and gently after that at S12A. The decrease was linear at S12B. There was almost no change in PO_4_–P before 2008, a decrease from 2008 to 2010, and almost no change in recent years at S12C. There were two and three small peaks—1993, 2000 at S12D and 1993, 2000, 2005 at S333. The PO_4_–P concentration did not change at S332. There was a distinct peak in PO_4_–P at S18C in 1995 which decreased after 2008. TP fluctuated; there was an upwards trend in 1985–1990, 1995–2000, and 2005–2008 and a downwards trend in 1990–1995 and 2000–2005 and after 2008 for S12 A to S12D. There was an upwards trend in TP from 1985 to 1990 and from 1995 to 2000, and a downwards trend from 1990 to 1995 and after 2000 at S333 and S332. TP decreased slowly from 1985 to 2005 at S18C.

### 3.3. Multivariate Statistical Analysis Results

#### 3.3.1. Cluster Analysis

**Figure 5:**
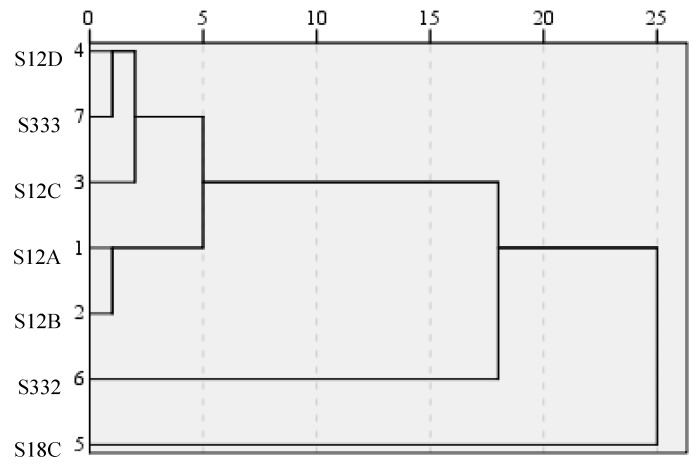
Dendrogram showing the clustering of sampling sites according to the water quality parameters of selected sites.

Based on the 10 variables, the seven sampling stations were classified by CA into four distinct clusters: A, B, C, and D (Figure 5). Cluster A was formed with S12A and S12B, Cluster B included S12C, S12D, and S333, while S332 and S18C were ascribed to Clusters C and D. The five stations located on the northern boundary were divided into two groups. S332 and S18C were divided separately. The seven stations can be reduced to four monitoring stations with the same monitoring function. Thus, more useful information could be obtained from as few stations as possible.

#### 3.3.2. Principal Component Analysis

**Figure 6:**
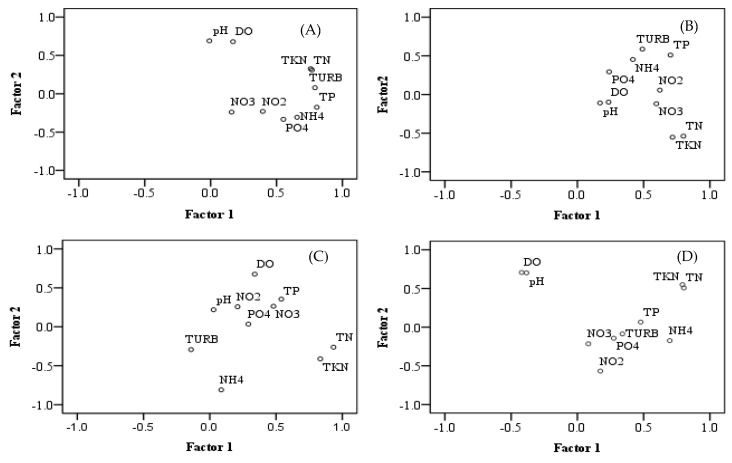
Factor loadings for the first factor (Factor 1) and the second factor (Factor 2). (**A**–**D**) indicate S12A, S12D, S332, and S18C respectively.

To identify the main pollution factors that influenced the identified group, S12A, S12D, S332, and S18C were selected as the typical stations for the PCA analysis (Figure 6). The four factors in Cluster A (S12A) explained 75.24% of the total variance. The first factor explained 33.91% of the total variance with heavy loading on TURB, TKN, TN and TP of 0.792, 0.759, 0.768, and 0.806, respectively. Factor 2 was dominated by DO and pH, accounting for 14.93% of the total variance. Factor 3 was moderately loaded by NO_2_, NO_3_, and PO_4_–P, explaining 13.40% of the total variance. For Cluster A, the turbidity induced by total nitrogen and phosphorus and other non-point sources was concluded to be main pollution parameter. For Cluster B (S12D), the four factors explained 69.95% of the total variance. The first factor explained 29.48% of the total variance; TN had strong loading and TP, TKN, NO_2_, and NO_3_ had moderate loading. Factor 2 was dominated by TURB, which accounted for 15.28% of the total variance. Factor 3 was moderately loaded by PO_4_–P, which accounted for 13.62% of the total variance. Nitrogen and phosphorus were identified as the main pollution parameters in Cluster B. For Cluster C (S332), the four factors explained 76.08% of the total variance. The first factor explained 23.63% of the total variance with TN and TKN loaded strongly. For Factor 2, NH_4_ was strongly loaded and DO was moderately loaded, accounting for 17.46% of the total variance. Factor 3 accounted for 13.24% of the total variance with moderately loaded pH and NO_2_. Nitrogen was the most dominant factor and phosphorus was the second most dominant factor in Cluster C (S332). For Cluster D (S18C), the four factors explained 72.74% of the total variance. The first factor explained 25.40% of the total variance with TN and TKN being strongly loaded and NH_4_–N being moderately loaded. Factor 2 was dominated by DO, pH, and NO_2_ which accounted for 19.82% of the total variance. Factor 3 was moderately loaded by PO_4_–P and TP which accounted for 15.69% of the total variance. The analysis indicated that TN and TP are the main pollution factors.

## 4. Discussion

### 4.1. Linkage of Water Quality Change with Precipitation

**Figure 7:**
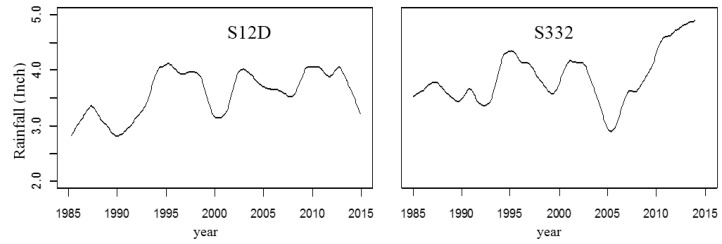
Rainfall trend at S12D and S332.

**Figure 8:**
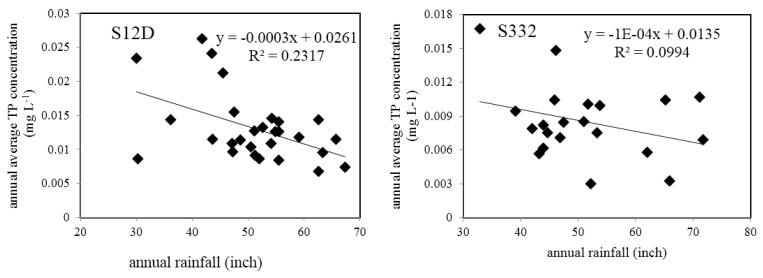
Linear regression between the annual average TP concentration and annual precipitation at S12D and S332.

There are only two seasons (dry season and wet season) in the study area. The water quality change trend was closely associated with rainfall, for example, S12D and S332. The monthly rainfall trend is illustrated in Figure 7. Increasing rainfall resulted in increasing flow which resulted in a decreasing concentration of TP. Figure 7 shows that the rainfall was low in 1990 and 2000, and the corresponding TP concentration peaked in those two years (Figure 4). In 1995, the rainfall was high and the TP concentration values at all stations were the lowest. The TP concentration was negatively correlated with rainfall. Linear regression between the annual average TP concentration and annual precipitation at S12D (from 1985 to 2014) and S332 (from 1985 to 2005) is illustrated in Figure 8. A significant negative correlation (*p* = 0.01) was observed at S12D. In the wet season, the chemistry and bioavailability of N and P in stormwater are subjected to further settling and biological degradation during the course of flow conveyance from agricultural fields to the basin outlet through the drainage canal network [23]. In this case, the catchment can serve as a functional physicochemical filter that regulates the fate and transport of nutrients [24]. Thus, the concentration of TP was lower in high flow years than low flow years. The concentration of N and P is also connected with the hydraulic residence time and land types and so on. In the area with high agricultural non-point sources, the TP concentration had a significant increasing trend with agricultural runoff in the wet season [25]. In this study area, the TP concentration declined in the wet season, which showed that the phosphorus content in the soil was low. There was no obvious correlation between rainfall and the PO_4_–P concentration. The Pearson correlation coefficients of water quality variables and monthly rainfall at S12D and S332 are shown in Table 3. DO and rainfall showed a significant negative correlation at the two stations (*p* = 0.01).

### 4.2. Correlation of Water Quality and Management

Water flows to the S12 structures through S333 and then enters the ENP. Water entering the L29 originates from WCA-3. There are many natural variations in geology, hydrology, and vegetation, and differences in water management and land uses in the two different regions which explains the differences in observed values between the northern and eastern structure groups. For example, water flowing into WCA-3 through the STA system resulted in improved water quality. The water inflowing from the eastern structures had a considerable residence time in the canals before entering the ENP, and land uses adjacent to these canals include agricultural and urban uses, which were managed by best management practices (BMPs) [15]. A possible reason for TN concentration not changing after a change in rainfall may be the change in management that occurred in the early 1990s. Non-point source pollution was effectively controlled after that. The temporal changes in nutrients are complex and affected by many factors (discharge, transport, and transformation processes), so it is not possible to completely explain them [26]. The TN concentration in water decreased sharply from 1985 to 1995 and decreased slowly after 1995 (Figure 3). TKN changed similarly to TN, which indicated that BMPs effectively reduced organic nitrogen. However, according to the PCA, nitrogen pollution control still cannot be ignored.

### 4.3. Management Implications 

From the trend analysis above, we inferred that organic nitrogen decreased at all stations, but the PCA analysis showed that nitrogen in water should still be considered to be a principal factor. The TP concentration was higher in the dry season than in the wet season. So, increasing the transfer water quantity is necessary to maintain water quality. Secondly, submersed macrophytes can absorb nitrogen and phosphorus in the water and sediment and increase the DO in the water. Thirdly, although the nitrogen and phosphorus are lower and the turbidity is relatively high in the eastern structure group, it is necessary to strengthen the role of the constructed ecological slope in the riparian buffer zones. The ecological slope protects the soil slope from collapsing, and vegetation on the slope can intercept the suspended solids in runoff and then reduce the turbidity in the water when it rains. Finally, to streamline administration, the seven stations can be reduced to four monitoring stations, and management practices should still focus on controlling nitrogen and phosphorus content in the future.

## 5. Conclusions

This paper demonstrated the temporal changes in water quality from seven monitoring stations at inflows to the ENP from 1985 to 2014 with multivariate statistical analysis. The STL method is perfectly adaptable to the water quality time series trend, and CA and PCA are useful tools for understanding the complex nature of water quality issues by identifying groupings in the set of data. We conclude that (i) the DO concentration range was 0.1–15.4 mg L^−1^ with a very clear downwards trend from 1990 to 1995 at the seven stations, and a correlation with rainfall. The pH values showed similar slight upwards trends at all stations, and the mean values were in the range of 7.19–7.45. All stations showed an obvious downwards trend in turbidity from 1990 to 1998, and there was an upwards trend at S12A, S12B, S12C, and S12D after 2005. (ii) The mean values of TN at S12C, S12D, and S333 were higher than the standard value, and TP exceeded this criterion at all stations except at S332 and S18C. The concentrations of TN at S12C, S12D, and S333 were relatively higher than at other stations. There was almost no change trend in NH_4_–N, but downwards trends in TKN and TN indicated that organic nitrogen decreased at all stations. The mean values of TP and PO_4_–P at both S332 and S18C in C111 were much lower than those at S333 in L31W. There was a downwards trend in PO_4_–P at all stations. The TP concentration was negatively correlated with rainfall. The increase in the TP concentration from 1985 to 1990 and from 1995 to 2000 may be attributed to the decline in precipitation. (iii) The cluster analysis suggested that seven inflow sites could be classified into four distinct clusters. This finding is useful for designing water quality monitoring programs that maximize the amount of variability captured in as few stations as possible. The principal component analysis showed that TP, and especially TN, are the main pollution factors. Therefore, management practices should still focus on controlling nitrogen and phosphorus contents in the future.

## Figures and Tables

**Table 1:** Analytical methods for selected water quality parameters

Water Quality Parameter	Detection Limit *	Analytical Method ^#^
DO	0.1 mg/L	EPA 360.1
pH	-	EPA 150.1
Turbidity	0.1 NTU ^@^	SM 2130B
NH_4_–N	0.008 mg/L	SM 4500-NH_3_H
NO_X_–N	0.004 mg/L	SM 4500NO_3_F
TKN	0.05 mg/L	EPA 351.2
PO_4_–P	0.002 mg/L	SM4500PF
TP	0.002 mg/L	SM 4500PF

^#^ EPA methods are from the USEPA; SM methods are from the American Public Health Association. * Detection limits could vary due to the improvement of laboratory techniques during the study period (1985–2014). ^@^ Nephelometric turbidity units.

**Table 2:** Basic statistics of the monthly measured data at seven stations

Parameters		S12A	S12B	S12C	S12D	S333	S332	S18C
DO (mg L^−1^)	Max	15.4	11.6	8.4	14	8.1	8.5	11.4
	Min	0.20	0.17	0.29	0.2	0.3	0.1	0.6
	Mean	4.40	5.79	3.89	3.70	3.59	3.42	5.17
	SD	1.65	1.72	1.57	1.66	1.47	1.95	1.47
pH	Max	9.71	8.95	9.26	8.85	8.52	8.76	8.89
	Min	5.80	5.90	5.80	5.90	6.00	6.00	5.35
	Mean	7.26	7.22	7.19	7.26	7.28	7.28	7.45
	SD	0.30	0.40	0.29	0.31	0.27	0.37	0.27
Turbidity (NTU)	Max	11.60	16.40	9.10	12.80	20.00	15.00	25.00
	Min	0.10	0.05	0.1	0.2	0.3	0.69	0.44
	Mean	1.51	1.44	1.37	1.63	1.7	2.57	1.82
	SD	1.45	1.19	1.06	1.53	1.92	1.76	1.92
NH_4_–N (mg L^−1^)	Max	1.00	1.36	0.65	0.49	0.35	0.5	0.49
	Min	0.003	0.003	0.005	0.005	0.005	0.005	0.005
	Mean	0.048	0.053	0.041	0.044	0.042	0.145	0.027
	SD	0.087	0.099	0.061	0.060	0.049	0.098	0.049
TKN (mg L^−1^)	Max	7.80	2.81	3.52	3.21	3.88	2.36	2.78
	Min	0.025	0.25	0.57	0.65	0.025	0.25	0.025
	Mean	1.14	1.15	1.17	1.30	1.34	0.84	0.67
	SD	0.55	0.52	0.51	0.32	0.33	0.36	0.33
Total nitrogen (TN) (mg L^−1^)	Max	7.85	2.81	3.53	4.4	5.25	3.28	2.95
	Min	0.11	0.25	0.59	0.73	0.79	0.26	0.25
	Mean	1.20	1.25	1.29	1.37	1.44	0.92	0.76
	S.D	0.59	0.59	0.40	0.36	0.41	0.41	0.41
PO_4_–P (mg L^−1^)	Max	0.062	0.057	0.134	0.037	0.037	0.02	0.017
	Min	0.001	0.001	0.001	0.001	0.001	0.002	0.001
	Mean	0.0037	0.0035	0.0038	0.0044	0.0038	0.0030	0.0026
	SD	0.0040	0.030	0.0086	0.0049	0.0046	0.0023	0.0046
TP (mg L^−1^)	Max	0.25	0.48	0.13	0.068	0.093	0.057	0.056
	Min	0.001	0.002	0.001	0.002	0.001	0.002	0.001
	Mean	0.021	0.017	0.013	0.014	0.015	0.0085	0.0072
	SD	0.14	0.094	0.015	0.0098	0.011	0.0062	0.011

**Table 3:** Pearson correlation coefficients of water quality variables and monthly rainfall at S12D and S332.

Water Quality		DO	pH	Turbidity	NO_3_–N	NO_2_–N	NH_4_–N	TKN	PO_4_–P	TN
S12D	Pearson Correlation	−0.202 **	0.089	0.066	0.014	0.138 *	−0.080	−0.030	0.048	−0.021
	Sig. (two-tailed)	0.001	0.147	0.282	0.822	0.023	0.192	0.621	0.434	0.728
S332	Pearson Correlation	−0.292 **	−0.121	0.158 *	−0.032	0.050	−0.003	−0.141 *	0.001	−0.137 *
	Sig. (two-tailed)	0.000	0.055	0.012	0.611	0.430	0.966	0.026	0.988	0.030

** Correlation is significant at the 0.01 level (two-tailed). * Correlation is significant at the 0.05 level (two-tailed).
